# Carbon Nanotube Driver Circuit for 6 × 6 Organic Light Emitting Diode Display

**DOI:** 10.1038/srep11755

**Published:** 2015-06-29

**Authors:** Jianping Zou, Kang Zhang, Jingqi Li, Yongbiao Zhao, Yilei Wang, Suresh Kumar Raman Pillai, Hilmi Volkan Demir, Xiaowei Sun, Mary B. Chan-Park, Qing Zhang

**Affiliations:** 1 School of Electrical and Electronic Engineering, Nanyang Technological University, Singapore 639798, Singapore; 2 School of Chemical and Biomedical Engineering, Nanyang Technological University, Singapore 639798, Singapore

## Abstract

Single-walled carbon nanotube (SWNT) is expected to be a very promising material for flexible and transparent driver circuits for active matrix organic light emitting diode (AM OLED) displays due to its high field-effect mobility, excellent current carrying capacity, optical transparency and mechanical flexibility. Although there have been several publications about SWNT driver circuits, none of them have shown static and dynamic images with the AM OLED displays. Here we report on the first successful chemical vapor deposition (CVD)-grown SWNT network thin film transistor (TFT) driver circuits for static and dynamic AM OLED displays with 6 × 6 pixels. The high device mobility of ~45 cm^2^V^−1^s^−1^ and the high channel current on/off ratio of ~10^5^ of the SWNT-TFTs fully guarantee the control capability to the OLED pixels. Our results suggest that SWNT-TFTs are promising backplane building blocks for future OLED displays.

With increasing demands for a variety of robust, light-weighted, and wearable electronic devices, the flexibility and transparency of the devices are required for next generation electronics such as flexible displays, sensors and photovoltaic systems, *etc*^1^,^2^,^3^. Recently, organic light-emitting diode (OLED) flexible displays have attracted a lot of attention. OLED displays possess many advantages over traditional liquid crystal displays (LCD), such as self-emission, high light efficiency, high brightness and contrast, wide viewing angle, low power consumption, and excellent flexibility, *etc*^4^,^5^. They have been used in curved televisions, cell phones, digital cameras and other mobile devices. These displays are driven by thin-film transistors (TFTs) whose channel materials are typically amorphous silicon, polycrystalline silicon, organic and metal oxide semiconductors, nanowires, *etc*. Amorphous silicon TFTs suffer from a low mobility (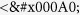
<1 cm^2^V^−1^s^−1^) and relatively low driving capacity^6^,^7^. As a replacement, polycrystalline silicon TFTs can provide a bit higher mobility and driving capacity^8^, but their relatively high cost, high-temperature processing, and optical opacity are not compatible with the requirements of future display electronics. In comparison, organic and metal oxide semiconductor TFTs have high optical transparency and can be processed at low-temperatures. But, similar to amorphous silicon TFTs, they have a relatively low device mobility^9^,^10^,^11^,^12^,^13^. Although OLED displays driven by In_2_O_3_ nanowire-based transistors have been reported^14^,^15^, relatively poor device uniformity, low reliability, and processing challenges still need to be overcome for good performances.

A single-walled carbon nanotube (SWNT) network inherits the unique properties of SWNTs^16^,^17^,^18^ and causes a high device uniformity due to the statistical averaging of multipath transports in the network. In addition, easy accessible fabrication process of SWNT network makes it more suitable to be integrated with scalable OLED pixels on a large-area substrate. Recently, SWNT-TFT based flexible devices^19^,^20^, integrated logic circuits^19^,^21^,^22^,^23^,^24^, and even a prototype of carbon nanotube-based computer^25^ have shown the outstanding electrical properties and excellent performance. SWNT-TFTs driver circuits for OLED displays have also been demonstrated on both flexible and hard substrates^1^,^26^,^27^,^28^,^29^,^30^. Due to the developments of high-performance TFTs using sorted semiconducting (sc)-SWNTs^27^,^31^,^32^,^33^, the most reported SWNT-TFTs driver circuits were made from sorted high-purity sc-SWNTs to achieve high on/off ratios^1^,^27^,^28^,^29^,^30^. Zhang *et al.* (Ref. 30) firstly demonstrated sorted sc-SWNTs driver circuits for an active matrix (AM) OLED display (20 × 25 pixels). As they employed the bottom gate structure and coated solution-based highly pure SWNTs onto the gate dielectric layer, they did encounter several technical challenges, say coating SWNTs uniformly in the channel regions and reducing the dielectric layer roughness, *etc*. In fact, they did not demonstrate the static and dynamic images of the display and ~30% of the pixels were not turned on. In addition, adoption of solution-based sorted sc-SWNTs is usually concomitant with several other problems, like contaminants of the surfactants and shortening of the nanotubes in the sorting and/or coating processes^22^,^34^. The local bottom-gate structure is usually employed in TFTs fabrication with solution-based separated SWNTs^27^,^29^. However, in the local bottom-gate configuration, the SWNTs channel is exposed to its environment. The conduction characteristics of the SWNT-TFTs are usually changed after passivation process. All these problems/challenges should be ticketed in order to develop high performance SWNT driver circuits for real static and dynamic AM OLED displays. However, to our knowledge, such SWNT driver circuits have not been reported yet.

In this paper, we demonstrate, for the first time, chemical vapor deposition (CVD)-grown random SWNT network based driver circuits for static and dynamic AM OLED (6 × 6 pixels) displays. The random SWNT network used here has very low contamination and very few short defective SWNTs (see Supplementary Fig. S1 online). With a top-gate structure, our SWNT-TFTs show an excellent uniform performance with the device mobility of ~45 cm^2^V^−1^s^−1^ and channel current on/off ratio of ~10^5^. These key parameters ensure a good control capability of the driver circuit to the large-scale OLED display.

Here, each AM OLED display pixel is integrated with two SWNT-TFTs and one capacitor (2T1C), as shown in Fig. 1a. The blue dashed box highlights a single unit 2T1C circuit which consists of one switching transistor (ST), one driving transistor (DT), one charge storage capacitor (*C*_S_), and one OLED pixel^35^. When the scan line (*V*_SCAN_) is selected, the ST is switched on so that the image information from the data line (*V*_DATA_) is written to the gate of the DT. Meanwhile, the image information, *i*. *e*. the voltage from the data line, is also stored and stabilized on *C*_S_ in one frame time, which is essential for the dynamic row-by-row scanning mode of present display technology. The DT is needed to drive each OLED pixel with the current from *V*_DD_. Figure 1b shows the optical image of the 2T1C structure. The total area of the single unit is 820 × 820 μm^2^, within which the OLED pixel occupies an area of 480 × 480 μm^2^, with an aperture ratio (defined as the ratio of the OLED pixel area to the single unit area) of 34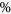. In principle, the aperture ratio could be further increased by optimizing layout designs and processing flows. Figure 1c shows a schematic cross-sectional perspective view of the single unit device structure fabricated on a quartz substrate with CVD-grown SWNT network channel, patterned Ti/Au (5 nm/30 nm) source and drain electrodes, Si_3_N_4_ (100 nm) gate dielectric, Ti/Au (10 nm/100 nm) top-gate electrodes, integrated green OLED, and a 300 nm SiO_2_ passivation layer.

To begin with, a uniform random SWNT network is grown on a quartz substrate on which ferritin has been deposited as catalyst^21^,^24^. Carbon feed stock into the thermal CVD is provided by flowing a mixture of H_2_ and Ar through an ethanol bubbler. The density of the SWNTs can be controlled through the density of ferritin and the flow rates of H_2_ and Ar gases. A field-emission scanning electron microscope (FE-SEM) image (Fig. 2a) of a randomly grown SWNT network suggests that, the average length of the as-grown SWNTs is more than 10 μm, far larger than the length (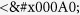
2 μm) of solution-based pre-separated SWNTs^27^,^28^. Probably because of the long SWNTs and small number of SWNT-SWNT contact junctions, the device mobility is larger than those reported TFTs made with solution-based separated SWNTs in Refs. 27 and 28. To reduce metallic SWNTs percolating pathways between the source and drain, the conventional striping technique^21^,^26^,^36^ is applied to pattern the random SWNT network into parallel strips with a width of 5 μm and a spacing of 4 μm, as shown in Fig. 2b. After the striping process, the SWNT-TFTs’ current on/off ratio is typically 10^5^.

Figure 3a shows the top-gate configuration of the SWNT-TFTs. On the quartz substrate with CVD-grown random SWNT network, 5 nm Ti/30 nm Au are deposited using an electron-beam (e-beam) evaporator as the source and drain electrodes. After the striping process, 100 nm Si_3_N_4_ gate dielectric is deposited by plasma enhanced chemical vapor deposition (PECVD), followed by patterning of the top-gate electrode (10 nm Ti/100 nm Au). The SWNTs outside the channel region are removed by oxygen plasma etching. Figure 3b shows the transfer (*I*_D_-*V*_G_) characteristics (the red curve is plotted in the log scale and the blue curve is in the linear scale) and transconductance (*g*_m_-*V*_G_) characteristics (the black curve) of a typical ST with a channel length of *L* = 80 μm and a channel width of *W* = 200 μm. The device shows a typical n-type behavior and the on-current of 11.7 μA at *V*_D _= 1 V and *V*_G _= 10 V. An on/off ratio greater than 10^5^ and the peak transconductance of 2.9 μS are obtained. It is known that SWNT-TFTs are typically p-type in ambient air, because of oxygen molecules or moisture adsorbed on the sidewalls of the SWNTs and/or SWNT-metal contacts^37^,^38^. However, in our top-gate configuration, n-type characteristics are generally observed mainly due to the desorption of oxygen molecules or moisture during deposition of Si_3_N_4_ gate dielectric^24^. Taking the electrostatic coupling between SWNTs^27^,^39^ into consideration, the device mobility can be determined to be ~45 cm^2^V^−1^s^−1^, far superior to that of conventional organic TFTs^9^ (~4 cm^2^V^−1^s^−1^) and amorphous silicon TFTs^6^ (<1 cm^2^V^−1^s^−1^). The output characteristics of a typical TFT are plotted in Fig. 3c with *V*_G_ varying from 10 to −10 V in steps of −5 V. As the transistor is fully turned off when *V*_G_ ≤ 0 V, the curves for *V*_G _= 0, −5, and −10 V are not distinguishable. Figure 3d summarizes the current on/off ratios and on-current values measured from all 36 STs in the driver circuit. The average values of the on-current and the on/off ratio are 11.83 μA and 4.9 orders of magnitude, with the standard deviations of 4.27 μA and 0.1 orders of magnitude, respectively. The small fluctuations suggest a highly uniform device performance among the 36 SWNT-TFTs.

After the driver circuit is fabricated, a 6 × 6 AM OLED pixel array is introduced. A single unit of the 2T1C layout is shown in Fig. 1a and Fig. 1b. Before introduction of the OLED pixels, a layer of 300 nm SiO_2_ is deposited to passivate and isolate the driver circuit from the OLED pixels, only leaving the pre-patterned ITO (used as the OLED anode) open in order to connect with the green OLED that is fabricated using the thermal evaporation technique. Figure 4a shows the optical image of a 6 × 6 driver circuit array before introduction of the OLED pixels. In order to confirm the control capability of each single unit circuit, the transfer (*I*_DD_-*V*_DATA_) characteristics are measured and shown in Fig. 4b. The scan line (*V*_SCAN_) is biased at 10 V to turn on the ST so that the DT can be controlled by the signal (*V*_DATA_) from the data line. An excellent on/off ratio (~10^5^) can be obtained. An on-current of 10 μA can be achieved when *V*_DD _= 1 V, *V*_DATA _= 10 V, and *V*_SCAN _= 10 V. This on-current is sufficient to power on an OLED pixel with the size of 480 μm × 480 μm. The current driving capability of the driver circuit is a very important factor for the OLED display.

The performance of the OLED pixel is evaluated after a standard Tris(8-hydroxy-quinolinato) aluminum (Alq_3_) green OLED with multilayer configuration is fabricated. The OLED is of a layered structure of ITO/MoO_3_/N,N’-Bis(naphthalen-1-yl)-N,N’-bis(phenyl)-benzidine (NPB)/Alq_3_/LiF/Al, as illustrated in Fig. 5a. The OLED shows an ideal diode-like characteristic with turn-on voltage of 2.7 V, as shown in Fig. 5b. At the turn-on voltage, the turn-on current for a 480 μm × 480 μm OLED pixel is 0.1 μA (the current density of 4.7 × 10^−4^ mA/mm^2^), much smaller than the on-current (~10 μA) of the single unit driver circuit (see Fig. 4b), suggesting that the single unit 2T1C driver circuit can fully drive an OLED pixel.

Finally, the AM OLED display with 6 × 6 pixels driven by 72 SWNT-TFTs is demonstrated. An external microcontroller unit (MCU) is used to generate the controlling signals *V*_SCAN_ and image signals *V*_DATA_. The controlling signals switch on the STs to enable the image signals to transfer to the gates of the DTs and turn on them to pass *I*_DD_ to further drive the OLED pixels. Figure 6a is a photo showing all 36 pixels are turned on when *V*_SCAN_ = 10 V, *V*_DATA _= 10 V, and *V*_DD _= 5 V are applied for all scan, data, and power lines. It can be seen that all pixels are turned on although some pixels emit relatively weak luminance. The weak luminance pixels are likely caused by the OLED quality degradation arising from surface roughness of the OLED anode (ITO)^40^. Another possible reason for the luminance difference could be the fluctuations of the on-current in the SWNT-TFTs (see Fig. 3d). To show the performance of this OLED display, program codes based on the row-by-row scanning mode which is widely employed in present display technology are developed and inputted into the external MCU to control the driver array. Figure 6b shows three letters “N”, “T”, and “U” on this OLED display in sequence. A video showing three running letters “N”, “T”, and “U” is provided in Supplementary online. To the best of our knowledge, this is the first demonstration of static and dynamic images implemented on an AM OLED display driven solely by a SWNT-TFT driver circuit.

In summary, we have successfully developed SWNT-TFT driver circuit for a 6 × 6 static and dynamic AM OLED display. CVD-grown random SWNT network is used as the channels of the top-gated TFTs to achieve a highly uniform performance, *i*. *e*. the channel current on/off ratio of ~10^5^ and device mobility of ~45 cm^2^V^−1^s^−1^. Our results confirm that the SWNT-TFT driver circuit is capable of controlling the OLED display. This work suggests that SWNT-based driver circuits could be of a great potential for future OLED displays.

## Methods

### Synthesis of random SWNT networks

The random SWNT networks used here are grown using thermal chemical vapor deposition on quartz substrates. The process flow is as follows: (1) A quartz substrate is cleaned by ultrasonication in acetone and IPA to remove organic contaminants and then dipped into a piranha solution (a 3:1 volumetric mixture of concentrated sulphuric acid to 30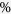 hydrogen peroxide solution) for 30 min to make the quartz surface extremely hydrophilic; (2) The catalyst solution of ferritin (Aldrich; diluted with de-ionized water at a volumetric ratio of 1:80) is spin-coated on the quartz substrate; (3) The quartz substrate is heated to 800 °C in a quartz tube to oxidize ferritin into iron oxide nanoparticles; (4) The quartz tube is then further heated to 925 °C in 100 s.c.c.m. hydrogen gas flow for 10 min to reduce the iron oxide to iron; (5) 30 s.c.c.m. argon gas and 15 s.c.c.m. hydrogen gas flow through an ethanol (carbon source) bubbler into the quartz tube while maintaining temperature (925 °C) for 15 min. The density of the random SWNTs is controllable through control of the concentration of ferritin solution and carrier gases (H_2_ and Ar) flow rates.

### Fabrication of top-gated SWNT-TFT driver circuit

First, on the quartz substrate with randomly as-grown SWNT network, the windows for the source and drain electrodes of the SWNT-TFTs are defined using standard photolithography (AZ 5214 as photoresist) and then 5 nm Ti and 30 nm Au are deposited using an electron-beam evaporation system. After a lift-off process, another photolithography process and oxygen plasma reactive-ion etching (200 mTorr, 20 s.c.c.m. O_2_ flow, 100 W radio frequency power) are applied to pattern the SWNT network into parallel strips to achieve a high on/off ratio of the SWNT-TFTs. In order to isolate the SWNT-TFTs from each other, oxygen plasma reactive-ion etching with the same conditions is employed again to clean up any SWNTs outside the channel regions which are protected by a patterned photoresist layer. Then, a 100 nm Si_3_N_4_ gate dielectric layer is deposited on the whole substrate by plasma enhanced chemical vapor deposition (PECVD). SiH_4_, NH_3_ and N_2_ (at the flow rates of 100, 20, 600 s.c.c.m., respectively) are used as the reaction gases and the radio frequency power is set at a low value of 20 W to minimize damage to the SWNT channels. With a high pressure of 1 Torr and a large N_2_ flow, the resultant plasma is cold and mild and the low plasma density results in a low deposition rate of 0.4 nm/s. After the gate dielectric deposition, the vias for interlayer interconnects are defined by photolithography and hydrofluoric (HF) acid etching (8 s in a 1:20 volumetric mixture of concentrated HF acid to de-ionized water). After via etching, the interlayer interconnects (10 nm Ti/90 nm Au) are deposited into the vias, followed by a lift-off process. Finally, the gate electrodes (10 nm Ti/100 nm Au) and interconnects (between the STs and the DTs) are patterned.

### 6 × 6 OLED pixels integrated with the top-gated SWNT-TFT driver circuit

Before OLED fabrication, the top-gated SWNT-TFT driver circuit is passivated with a 300 nm SiO_2_ layer, leaving only the OLED anode area open in each unit. After passivation, 300 nm indium-tin oxide (ITO) is deposited as OLED anodes using a radio frequency sputtering system. Then, a standard Tris(8-hydroxy-quinolinato) aluminum (Alq_3_) green OLED with structure of ITO/MoO_3_ [5 nm]/NPB [80 nm]/Alq_3_ [60 nm]/LiF [1 nm]/Al [150 nm] is fabricated using a thermal evaporator.

### Device and circuit characterizations

The morphology of random SWNT networks is characterized using a field-emission scanning electron microscopy (LEO 1550 Gemini SEM). The direct-current measurements of SWNT-TFTs and single unit driving circuit are carried out in air using a semiconductor parameter analyzer (Agilent, B1500A). The performances of OLED pixels are measured using LS-110 luminance meter (Konica Minolta). An external microcontroller unit (Arduino Duemilanove ATmega328) is used to input controlling and image signals to the SWNT-TFT driver circuit to create static and dynamic images on the OLED display.

## Additional Information

**How to cite this article**: Zou, J. *et al.* Carbon Nanotube Driver Circuit for 6 × 6 Organic Light Emitting Diode Display. *Sci. Rep.* **5**, 11755; doi: 10.1038/srep11755 (2015).

## Supplementary Material

Supplementary Information

Supplementary Movie S1

## Figures and Tables

**Figure 1:**
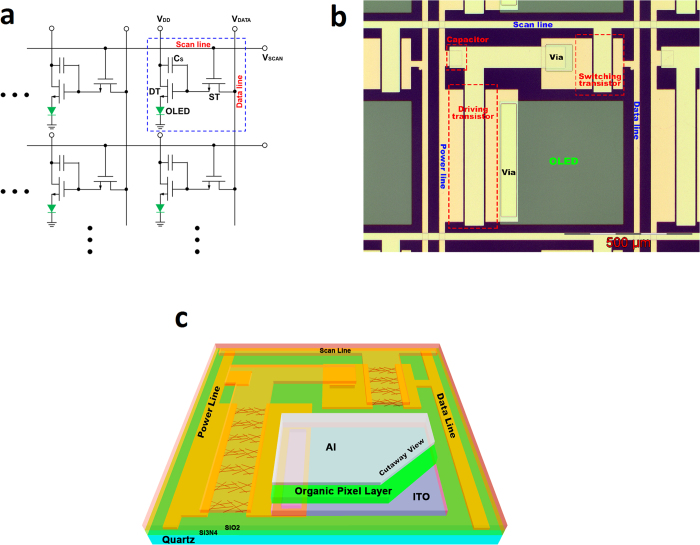
Structure of the AM OLED driver circuit design and layout. (**a**) Schematic diagram of AM OLED display design based on a 2T1C single unit circuit, consisting of one ST, one DT, one *C*_S_, and one OLED pixel. (**b)** Optical image of a single AM OLED display unit. The total area of the single unit is 820 × 820 μm^2^ including an OLED pixel area of 480 × 480 μm^2^. **(c)** A schematic cross-sectional perspective view of the single AM OLED display unit fabricated on a quartz substrate with CVD-grown SWNT network as the active channel, patterned Ti/Au source and drain electrodes, Si_3_N_4_ gate dielectric, Ti/Au top-gate electrode, integrated green OLED, and a SiO_2_ passivation layer.

**Figure 2:**
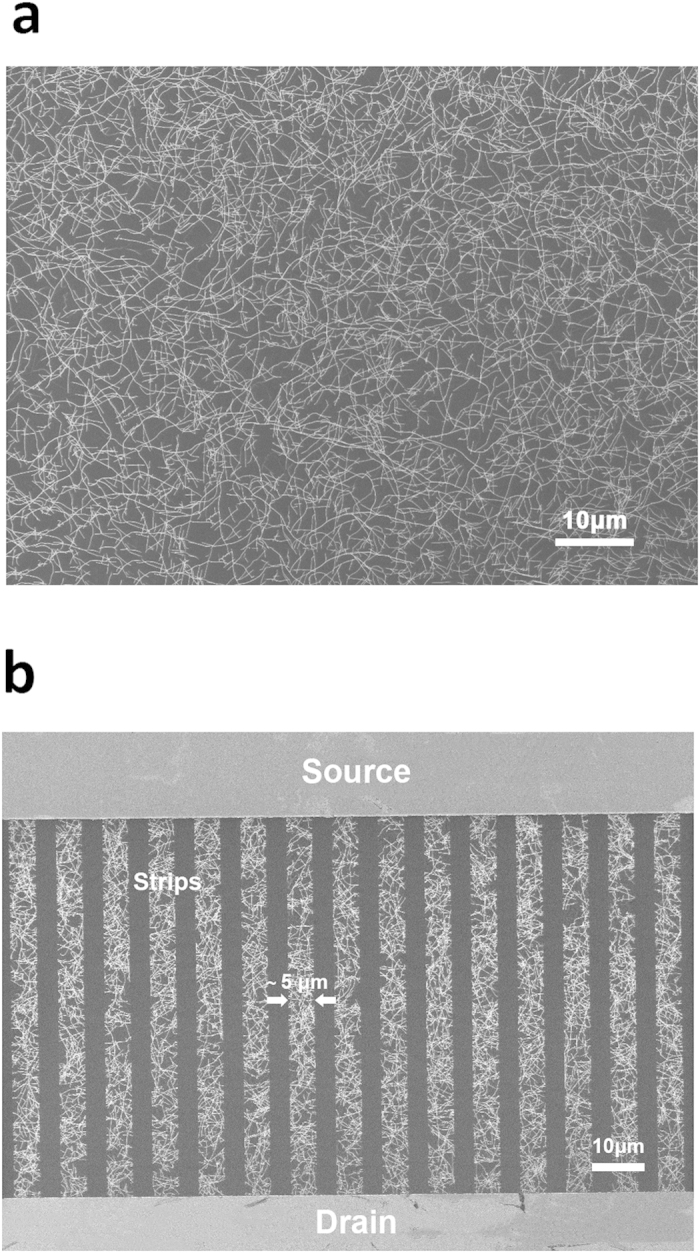
Field-emission scanning electron microscope images of SWNT network. (**a**) A CVD as-grown random SWNT network on a quartz substrate. **(b)** Parallel SWNT strips with a width of 5 μm.

**Figure 3:**
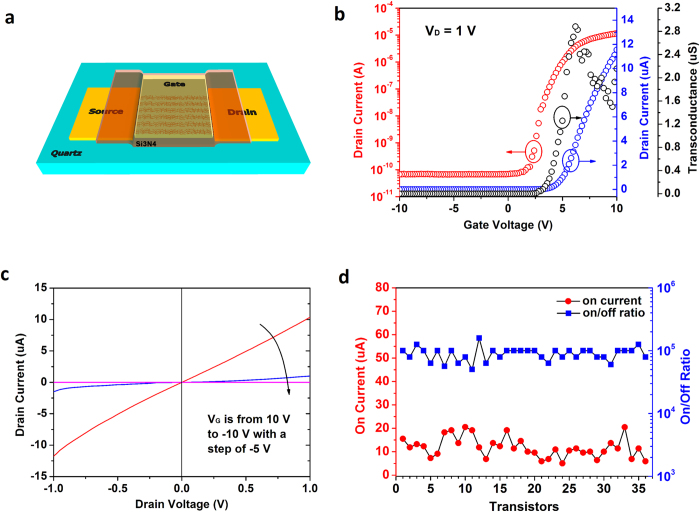
Electrical properties of top-gated SWNT-TFTs used in the AM OLED driver circuit. (**a**) Schematic diagram of the top-gated SWNT-TFT fabricated on a quartz substrate with Ti/Au source and drain electrodes, Si_3_N_4_ gate dielectric, and Ti/Au top gate. (**b**) Transfer (*I*_D_-*V*_G_) characteristics (the red curve is plotted in the log scale and the blue curve is in the linear scale) and transconductance (*g*_m_-*V*_G_) characteristics (the black curve) of a typical ST (channel length *L *= 80 μm, channel width *W* = 200 μm) with *V*_D _= 1 V. **(c)** Output (*I*_D_-*V*_D_) characteristics of the same transistor with *V*_G_ varying from 10 to −10 V with −5 V steps. **(d)** The current on/off ratios and on-current values of 36 STs (*L* = 80 μm, *W* = 200 μm).

**Figure 4:**
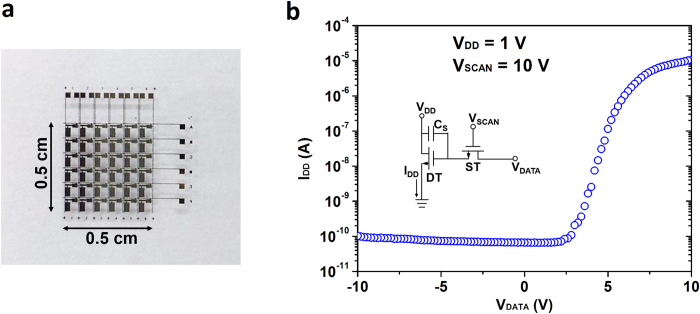
The SWNT-TFTs driver array with 2T1C configuration in each unit. (**a**) Photograph of the 6 × 6 driver array (0.5 × 0.5 cm^2^) before introduction of OLED pixels. (**b**) Typical transfer (*I*_DD_-*V*_DATA_) characteristics of a single unit 2T1C circuit when *V*_SCAN _= 10 V, *V*_DD _= 1 V. Inset: schematic diagram of the single unit 2T1C circuit.

**Figure 5:**
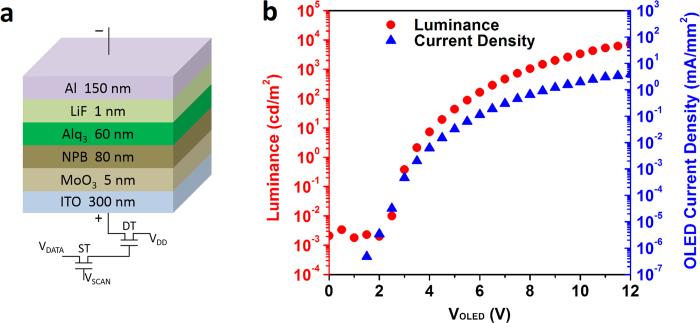
Structure and characteristics of a standard Alq3 green OLED. (**a**) Schematic diagram of the green OLED with the structure of ITO/MoO_3_/NPB/Alq_3_/LiF/Al. (**b**) The OLED luminance and current density versus the applied voltage on the OLED.

**Figure 6:**
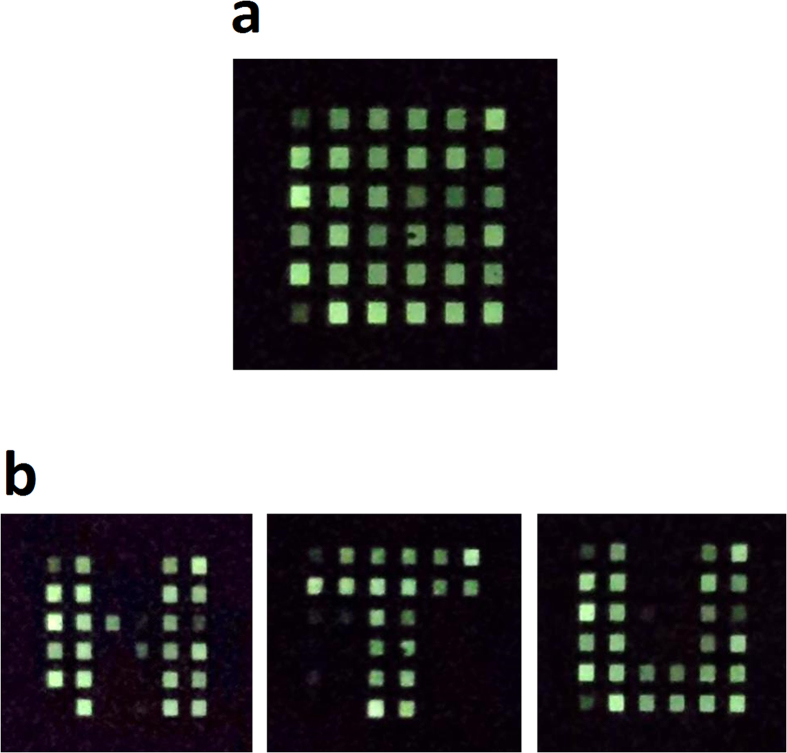
Display demonstration of the 6 × 6 AM OLED pixels driven by 72 SWNT-TFTs. (**a**) A photo showing the 36 pixels turned on under *V*_SCAN _= 10 V, *V*_DATA_ = 10 V, and *V*_DD _= 5 V. **(b)** The letters “N”, “T”, and “U” are displayed sequentially on this OLED display. A video showing three running letters “N”, “T”, and “U” can be found in Supplementary online.
