# Supplementary material for: Carbon Nanotube Driver Circuit for 6 × 6 Organic Light Emitting Diode Display
Source: Sci Rep. 2015 Jun 29;5:11755. doi: 10.1038/srep11755 (PMC4484243; doi:10.1038/srep11755)
Supplement: Supplementary Information [file srep11755-s1.pdf]

Carbon Nanotube Driver Circuit for 6 × 6 Organic Light Emitting Diode Display

Jianping Zou[1], Kang Zhang[1], Jingqi Li[1†], Yongbiao Zhao[1], Yilei Wang[2], Suresh Kumar Raman Pillai[2], Hilmi Volkan Demir[1], Xiaowei Sun[1*], Mary B. Chan-Park[2*] and Qing Zhang[1*]

[1]School of Electrical and Electronic Engineering, Nanyang Technological University, Singapore 639798, Singapore.

[2]School of Chemical and Biomedical Engineering, Nanyang Technological University, Singapore 639798, Singapore.

*e-mail: eqzhang@ntu.edu.sg; mbechan@ntu.edu.sg; exwsun@ntu.edu.sg

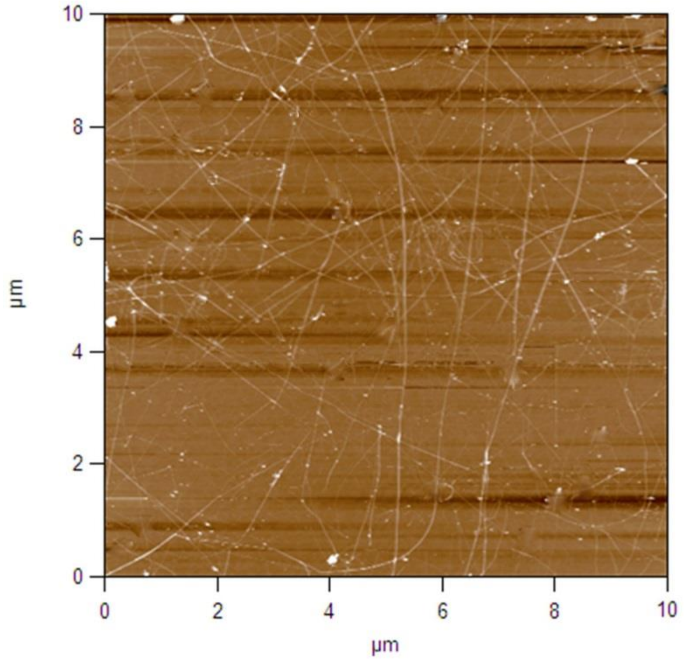

Figure S1. AFM image of CVD-grown SWNT network on quartz substrate.
